# A nomogram combining CT-based radiomic features with clinical features for the differentiation of benign and malignant cystic pulmonary nodules

**DOI:** 10.1186/s13019-024-02936-z

**Published:** 2024-06-27

**Authors:** Yi Yao, Yanhui Yang, Qiuxia Hu, Xiaoyang Xie, Wenjian Jiang, Caiyang Liu, Xiaoliang Li, Yi Wang, Lei Luo, Ji Li

**Affiliations:** 1 Department of Cardiothoracic Surgery, The First People’s Hospital of Neijiang, No. 1866, West Section of Hanan Avenue, Shizhong District, Neijiang, Sichuan 641000 China; 2 Department of Obstetrics and Gynecology, The First People’s Hospital of Neijiang, No. 1866, West Section of Hanan Avenue, Shizhong District, Neijiang, Sichuan 641000 China

## Abstract

**Background:**

Currently, the differentiation between benign and malignant cystic pulmonary nodules poses a significant challenge for clinicians. The objective of this retrospective study was to construct a predictive model for determining the likelihood of malignancy in patients with cystic pulmonary nodules.

**Methods:**

The current study involved 129 patients diagnosed with cystic pulmonary nodules between January 2017 and June 2023 at the Neijiang First People’s Hospital. The study gathered the clinical data, preoperative imaging features of chest CT, and postoperative histopathological results for both cohorts. Univariate and multivariate logistic regression analyses were employed to identify independent risk factors, from which a prediction model and nomogram were developed. In addition, The model's performance was assessed through receiver operating characteristic (ROC) curve analysis, calibration curve analysis, and decision curve analysis (DCA).

**Results:**

A cohort of 129 patients presenting with cystic pulmonary nodules, consisting of 92 malignant and 37 benign lesions, was examined. Logistic data analysis identified a cystic airspace with a mural nodule, spiculation, mural morphology, and the number of cystic cavities as significant independent predictors for discriminating between benign and malignant cystic lung nodules. The nomogram prediction model demonstrated a high level of predictive accuracy, as evidenced by an area under the ROC curve (AUC) of 0.874 (95% CI: 0.804–0.944). Furthermore, the calibration curve of the model displayed satisfactory calibration. DCA proved that the prediction model was useful for clinical application.

**Conclusion:**

In summary, the risk prediction model for benign and malignant cystic pulmonary nodules has the potential to assist clinicians in the diagnosis of such nodules and enhance clinical decision-making processes.

## Introduction

Lung cancer is the main cause of cancer-associated deaths worldwide due to its increasing incidence and mortality [1]. With the popularity of multi-detector CT scanners, a special pulmonary nodule has attracted widespread attention. Its CT manifestations include the inflatable cavity in the lung. Moreover, when the wall thickness is < 4 mm, the peripheral cavity is often affected by common signs of lung cancer, which are called cystic pulmonary nodules. Another study has defined cystic lung nodules as lesions with a wall thickness of < 4 mm and approximately 75% of their circumference; these histopathologically confirmed lung cancer cases are referred to as cystic lung cancer cases [2]. Although this type of lung cancer was first reported by Womack et al. [3] in 1941, subsequent studies have reported similar findings. Because of the limited sample size, the understanding of such lesions is insufficient, and they can be easily missed when making a diagnosis in clinical practice. Data from the International Early Lung Cancer Screening Program revealed that cystic lung cancer was found in approximately 3.7% of all cancer cases [4]. Low-dose CT is an efficient solution for the lung cancer screening and mortality reduction [5]. Nevertheless, distinguishing the characteristics of thin-walled cavities of cystic lung cancer on CT from those of benign diseases such as emphysema and pulmonary bullae is very difficult. Simultaneously, obtaining histopathological specimens of cystic pulmonary nodules by puncture biopsy is difficult. Thus, qualitative diagnosis is still a major challenge faced by thoracic surgeons and can result in misdiagnoses or delayed diagnoses of early lung cancer with cystic air cavities [6]. The Nederlands-Leuvens Longkanker Screenings Onderzoek lung cancer screening program reported that cystic lung cancer patients accounted for approximately 22.7% of all missed diagnoses [7]. Misdiagnosis and delayed diagnosis might cause early-stage lung cancer to progress to advanced disease or even tumor metastasis, with a 5-year survival rate of 5.3% [8]. Thus, a noninvasive method for distinguishing benign and malignant cystic pulmonary nodules has significant implications for clinicians [9]. To date, many diagnostic prediction models, including the most classic models, such as Mayo [10], VA [11], Brock [12], and PKUPH [13], have been proposed for non-cystic lung nodules. However, there is no diagnostic prediction model for detecting cystic pulmonary nodules. Thus, the present study aimed to establish a predictive model and nomogram for diagnosing cystic pulmonary nodules. Apart from determining various cystic pulmonary nodules, this nomogram might assist thoracic surgeons in making more reasonable clinical decisions as well as in achieving early prevention and timely intervention for high-risk nodules.

### Patient selection

We included patients with cystic pulmonary nodules who were admitted to the Thoracic Surgery Department of Neijiang First People’s Hospital from January 2017 to June 2023. The inclusion criteria were as follows: (1) had CT features reported as cystic cavities at a single or multiple time points and (2) underwent surgery or percutaneous lung biopsy to confirm the pathology. The exclusion criteria were as follows: (1) had cavities secondary to central necrosis of the previous solid lesions and (2) had lung cysts that could not be distinguished from surrounding emphysema, bronchiectasis, or cystic interstitial lung disease [7, 9].

### Data collection and variable definitions

Patient data were obtained from the medical records database of Neijiang First People’s Hospital. The study parameters were as follows: (1) demographic data: sex, age, history of lung disease, and tumor history; (2) tumor marker date: a carcinoembryonic antigen (CEA) level ≤ 5.09 ng/mL was considered negative, and > 5.09 ng/mL was considered positive; (3) CT imaging features: nodule diameter, mural nodule, vascular penetration sign, mural components, spiculation, pleural tag sign, mural morphology and thickness as well as the number of cystic cavities; and (4) histopathological results after surgery or lung puncture biopsy. The clinical data was collected well including blood samples after the patients had fasted on the morning of the second day of hospitalization. All chest CT scans were carried out in the supine position. Two experienced radiologists obtained subsequent CT readings, and any disputes were resolved by another senior radiologist after consultation. During CT feature acquisition, the nodule diameter was indicated by cross-sectional measurements across the largest diameter of the cyst [14]. The solid components on the wall of the cystic airspace indicated mural nodules. The vascular penetration sign was defined as the presence of a vessel crossing the node found on CT images [15]. The mural components were divided into two classes: nonsolid and solid [14]. Spiculation was defined as extension of the strands from the nodal margins into the lung parenchyma without touching the pleural surface [15]. A pleural tag indicated that the visceral pleura was pulling toward the pulmonary nodule [16]. The cyst’s inner surface was categorized as regulated or unregulated. Walls with thicknesses < 2 mm and ≥ 2 mm were denoted as thin and thick walls, respectively. Based on the number of septa, the cyst locules were classified as unilocular or multilocular [14].

### Establishment and evaluation of the predictive model

All factors were included in the univariate analysis. Additionally, all factors with *p* values < 0.05 in the univariate analysis were included in subsequent multivariate logistic regression analysis. The nomogram prediction model were established with R statistical software (Windows version 4.2.1), and the independent risk factors were included in the multivariate analysis. Hence, ROC curves were used to evaluate the identification efficiency of the predictive nomogram [17]. In addition, a nomogram calibration curve was generated to evaluate the disparities between the predicted probabilities and the actual results. Furthermore, decision curve analysis (DCA) was used to evaluate the clinical utility of the predictive nomogram according to the net benefits at different threshold probabilities [18].

### Statistical analysis

All the statistical analyses were carried out with SPSS 26.0 (IBM, Armonk, NY, USA) and R statistical (version 4.2.1) software. Normally distributed continuous variables are presented as the mean ± standard deviation (x ± SD). An independent sample t-test was used for intergroup comparisons. Moreover, the non-normally distributed continuous variables are presented as medians (interquartile ranges), and the Mann–Whitney U test was used to compare the two groups. Categorical variables are presented as cases and percentages, and comparisons between groups were carried out with either Pearson’s chi-square test (for *n* > 5) or Fisher’s exact test. Bilateral *p* values < 0.05 were considered to indicate statistical significance.

## Results

### Patient characteristics

**Fig. 1:**
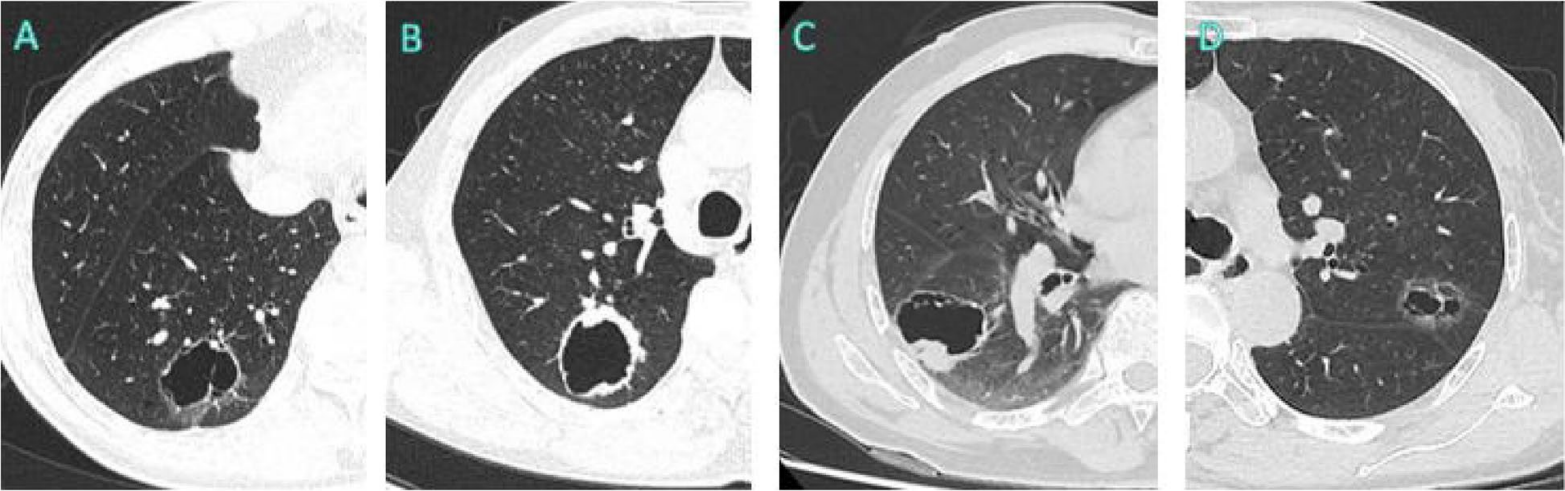
CT images of four morphological patterns of cystic pulmonary nodules. **A** Type I, mean thin wall(< 2 mm); **B** type II, mean thick wall(≥ 2 mm); **C** type III: a cystic airspace with a mural nodule; **D** type IV, tissue intermixed within clusters of cystic airspaces

Our study included 129 patients with cystic pulmonary nodules admitted to the Department of Thoracic Surgery of Neijiang First People’s Hospital from January 2017 to June 2023. Among them, 92 patients in the malignant group had 79 adenocarcinomas (10 adenocarcinomas in situ, 29 minimally invasive adenocarcinomas, and 40 invasive adenocarcinomas), 10 had squamous carcinomas, 2 had small cell lung cancer, and 1 had large cell lung cancer cases. Some cases are shown by typing in Fig. 1 [14]. The 37 patients in the benign group had 22 bronchial adenomas, 9 pulmonary bullae, 2 pulmonary tuberculosis cases, and 4 bacterial or fungal infections. The characteristics of the two groups in terms of malignancy and benign nature are shown in Table 1. There were no significant differences in sex, age, history of lung disease, tumor history, CEA level, lesion diameter, vascular penetration sign, mural components, pleural tag sign or mural thickness between the two groups (*P* > 0.05). Significant differences were observed in the mural nodules, spiculation, mural morphology, and number of cystic cavities between the two groups (*p* < 0.05).

**Table 1:** Clinical characteristics of patients with benign and malignant cystic pulmonary nodules

Characteristics	Malignancy (*n* = 92)	Benign (*n* = 37)	χ^2^/t	*P*
Sex (Male/Female)	45/47	19/18	0.063	0.862
Age (years, ± SD)	58.62 ± 11.66	57.57 ± 13.43	0.443	0.658
History of lung disease (Yes/No)	21/71	5/32	1.422	0.233
History of tumor (Yes/No)	4/88	5/32	3.416	0.065
CEA (Positive/Negative)	50/42	17/20	0.746	0.388
Nodule’s diameter (mm, ± SD)	18.74 ± 0.91	19.49 ± 0.85	0.429	0.669
Mural nodule (Yes/No)	61/31	17/20	4.585	0.032
Vascular penetration sign (Yes/No)	56/36	20/17	0.506	0.477
Mural component (non-solid/solid)	38/54	21/16	2.539	0.111
Spiculation sign (Yes/No)	67/25	14/23	13.826	< 0.001
Pleural tag sign (Yes/No)	49/43	13/24	3.473	0.062
Mural morphology (irregularity/regulation)	64/28	11/26	17.205	< 0.001
Mural thickness (≥ 2/ < 2mm)	50/42	19/18	0.095	0.758
Number of cystic cavity (multilocula/unilocular)	58/34	16/21	4.230	0.040

*CEA* carcinoembryonic antigen, *SD* standard deviation

### Univariate and multivariate analyses

Univariate and multivariate logistic regression analyses were performed (Table 2). Univariate analysis revealed that the presence of mural nodules (odds ratio [OR] = 2.315; 95% confidence interval [CI] = 1.064–5.039; *P* = 0.034), mural components (OR = 0.447; 95% CI = 0.206–0.970; *P* = 0.042), spiculation signs (OR = 4.403; 95% CI = 1.963–9.875; *P* < 0.001), mural morphology (OR = 5.403; 95% CI = 2.348–12.429; *P* < 0.001), and the number of cystic cavities (OR = 2.239; 95% CI = 1.030–4.865; *P* = 0.042) were potential risk factors. Subsequent multifactor analysis revealed that mural nodules (OR = 5.168; 95% CI = 1.797–14.864; *P* = 0.002), spiculation signs (OR = 5.771; 95% CI = 2.155–15.453; *P* < 0.001), mural morphology (OR = 7.501; 95% CI = 2.669–21.080; *P* < 0.001), and the number of cystic cavities (OR = 3.076; 95% CI = 1.132–8.353; *P* = 0.028) were independent predictors for distinguishing benign and malignant cystic pulmonary nodules, according to the *p* < 0.05 test.

**Table 2:** Univariate and multivariate logistic regression analyses

Characteristics	Univariate analysis		Multivariate analysis	
	OR (95%CI)	*P* value	OR (95%CI)	*P* value
Sex	0.907 (0.423–1.946)	0.802		
Age	1.007 (0.976–1.040)	0.656		
History of lung disease	1.893 (0.655–5.468)	0.238		
History of tumor	0.291 (0.074–1.151)	0.079		
CEA	1.401 (0.651–3.012)	0.389		
Nodule’s diameter	0.911 (0.598–1.389)	0.666		
Mural nodule	2.315 (1.064–5.039)	0.034	5.168 (1.797–14.864)	0.002
Vascular penetration sign	1.642 (0.761–3.542)	0.206		
Mural component	0.447 (0.206–0.970)	0.042		
Spiculation sign	4.403 (1.963–9.875)	< 0.001	5.771 (2.155–15.453)	< 0.001
Pleural tag sign	2.104 (0.955–4.633)	0.065		
Mural morphology	5.403 (2.348–12.429)	< 0.001	7.501 (2.669–21.080)	< 0.001
Mural thickness	1.128 (0.525–2.422)	0.758		
Number of cystic cavity	2.239 (1.030–4.865)	0.042	3.076 (1.132–8.353)	0.028

*OR* odds ratio, *CEA* carcinoembryonic antigen

### Nomogram establishment

**Fig. 2:**
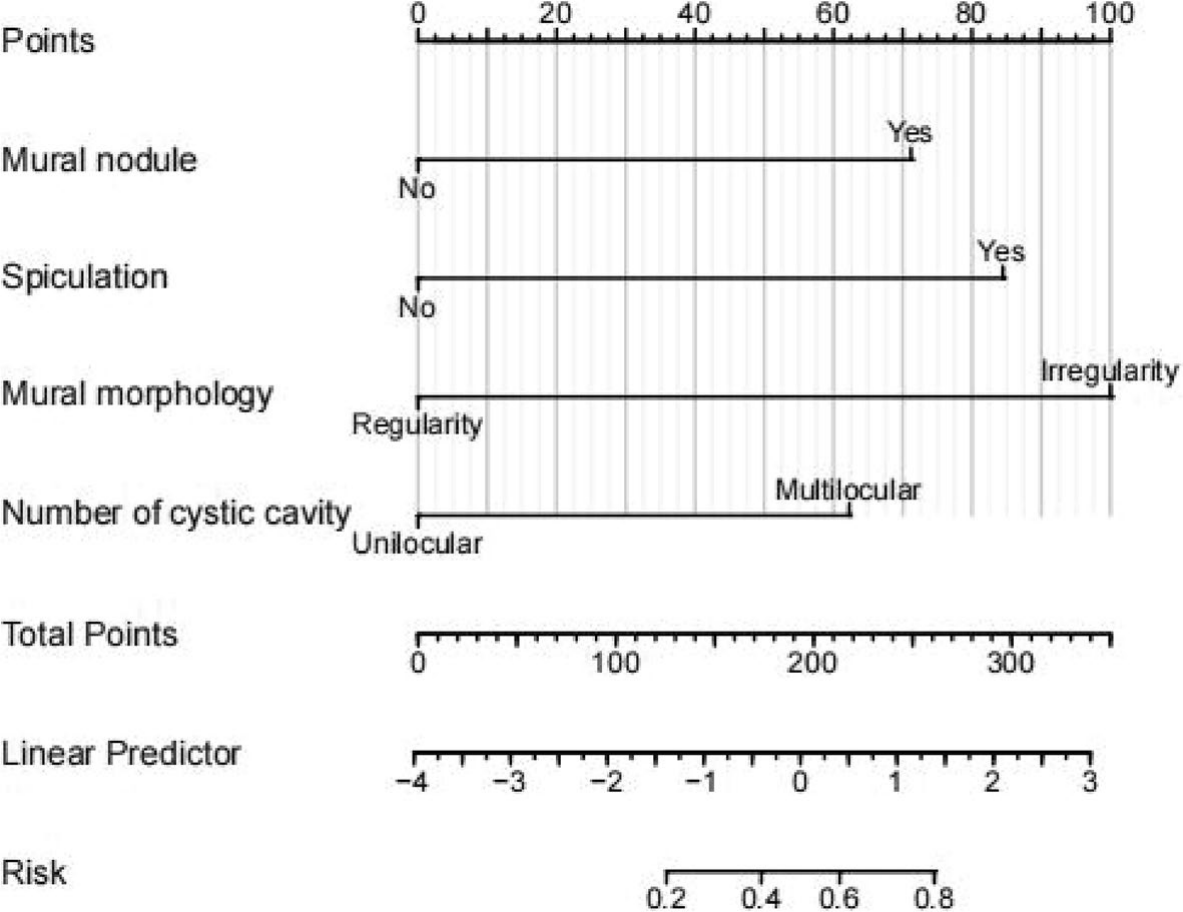
A nomogram for predicting the probability of malignancy in cystic pulmonary nodules. There are a total of 7 axes. Axes 2–5 represent four independent risk factors. An estimated score for axes 2–5 can be calculated by corresponding to the first axis, and the four scores are summed to the total score. The total point axis is subsequently used to predict the probability of malignancy for cystic pulmonary nodules

We constructed a nomogram of benign and malignant cystic pulmonary nodules according to four independent risk factors: mural nodules, spiculation, mural morphology, and the number of cystic cavities (Fig. 2). The corresponding scores were calculated from these four factors; the scores were further aggregated and projected onto the total subscale. Finally, total score calculated the probability of cystic pulmonary cancer to guide clinicians in further treatment.

### The predictive performance and validation of the nomogram

**Fig. 3:**
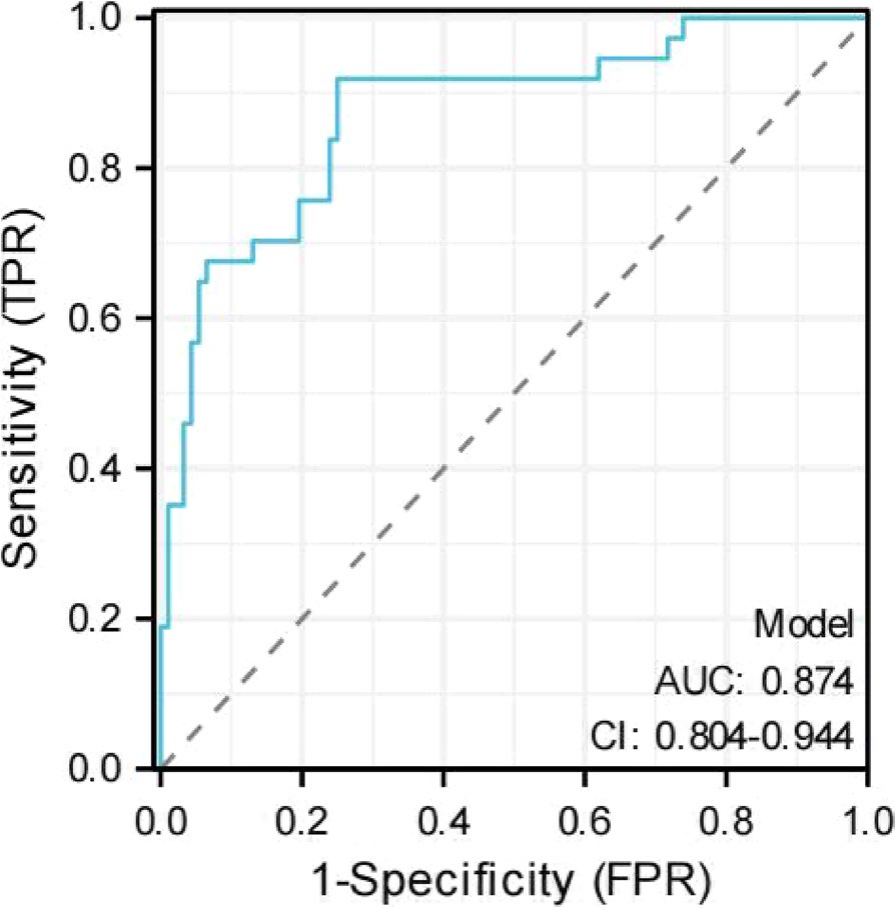
ROC curves of the nomogram for predicting the malignancy of cystic pulmonary nodules. ROC, receiver operating characteristic; AUC, area under the ROC curve

**Fig. 4:**
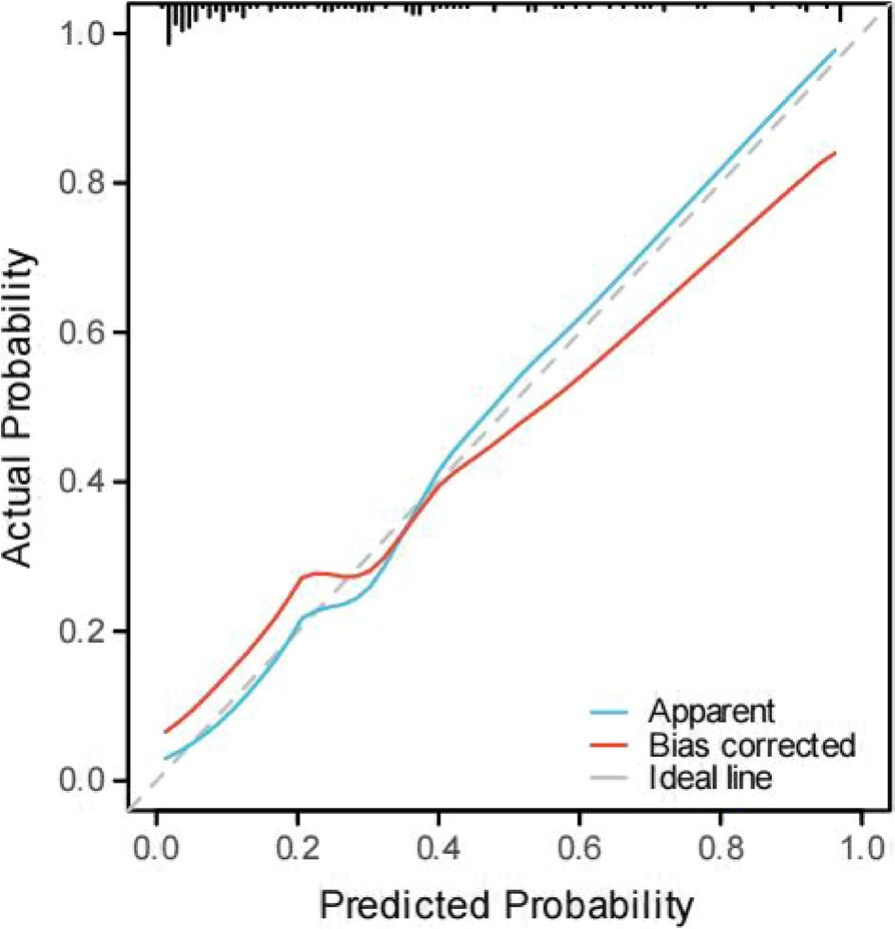
Calibration curves of the prediction nomogram.The X-axis represents the nomogram-predicted probability, and the Y-axis represents the actual probability of cystic pulmonary nodules. The gray dashed line represents the ideal curve. The blue line represents the apparent curve (non-corrected), and the red line represents the bias-corrected curve

**Fig. 5:**
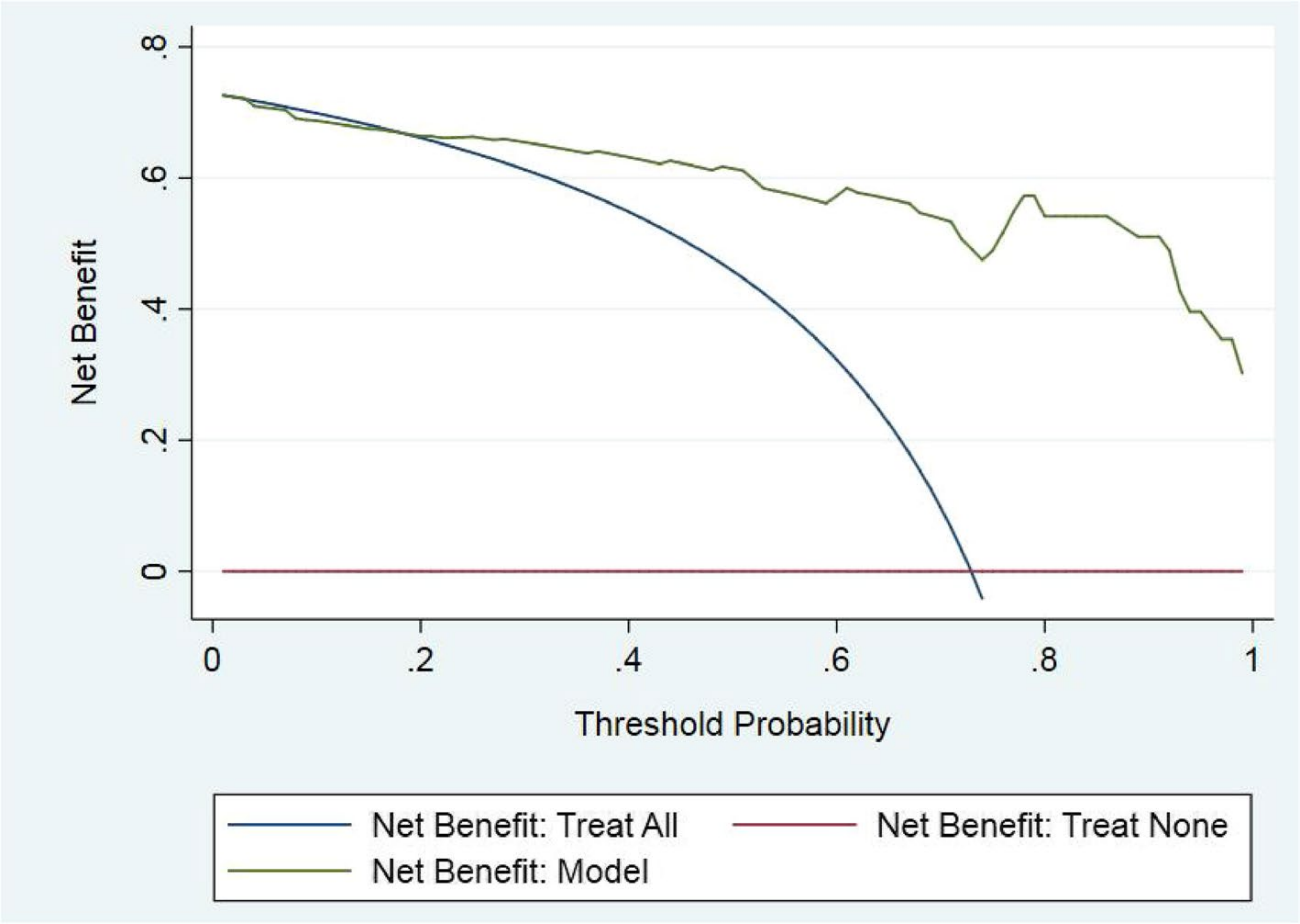
Decision curve analysis of the prediction nomogram. The X-axis represents the threshold probability and the Y-axis represents the net gain. The red line represents the assumption that all cystic pulmonary nodules are benign. The blue line represents the assumption that all cystic pulmonary nodules are malignant. The green line represents the predicted model

The ROC curve was used to assess the discriminative ability of the nomogram (Fig. 3). The area under the ROC curve (AUC) was 0.874 (95%CI: 0.804–0.944), suggesting that the nomogram had good prediction accuracy. Subsequently, the calibration power was evaluated with calibration plots. The calibration curve suggested good calibration of the predictive nomogram (Fig. 4). DCA was used to assess the clinical utility of the predictive nomogram (Fig. 5). The results showed that the model is suitable for clinical practice because the nomogram provided greater net benefit and broader threshold probabilities for predicting the risk of malignancy in cystic pulmonary nodules.

## Discussion

Currently, lung cancer is the most common cause of cancer-related deaths worldwide [1, 19]. Since a majority of lung cancers are detected in advanced stages, they have a poor prognosis. It is important to distinguish cystic pulmonary nodules from benign diseases such as emphysema and pulmonary vesicles to optimize treatment success. Hence, the lack of awareness regarding the features of cystic pulmonary cancer among clinicians has led to misdiagnosis as “alveolar wall thickening”. This figure accounts for 22.7% of all missed diagnoses in retrospective studies that included lung cancer screening tests [7]. Early lung cancer with cystic air cavities is often misdiagnosed, and its treatment is delayed [6]; moreover, its prognosis is worse than that of non-cystic lung cancer [20]. Several guidelines and consensuses have suggested that clinicians use a prediction model for malignancy probability to provide a reference for clinically identifying malignancies and invasive lesions [21]. Several prediction models, such as the Mayo model [10], the VA model [11], the Brock model [12], and the PKUPH model [13], have been designed for evaluating benign and malignant pulmonary nodules. Partial prediction models have incorporated advanced quantitative imaging methods, including CT attenuation, tumor diameter growth rate, and imaging omics [15, 22, 23]. However, all these classical models have certain limitations. Since the prospective Mayo model data were described > 20 years ago, the limited follow-up time could not fully clarify the nature of the nodules, resulting in poor timeliness and low accuracy. Furthermore, the VA model has lost its clinical significance because it is based on X-ray data. The PKUPH model did not define the time point of CT selection. However, in advanced radiomic models, the data analysis of the imaging omics model is complicated, and the model has poor clinical accessibility. Moreover, all these prediction models included only non-cystic pulmonary nodules and not cystic pulmonary nodules. Thus, our study focused on developing a clinical prediction model and a nomogram with good predictive performance for evaluating benign and malignant cystic pulmonary nodules. Consequently, our nomogram can be further employed to estimate the probability of malignant nodules in individuals with cystic pulmonary nodules can help clinicians make accurate clinical decisions.

Sex, age, history of lung disease, tumor history, CEA level, nodule diameter, mural nodule, vascular penetration sign, mural components, spiculation, pleural tag sign, mural morphology, mural thickness, number of cystic cavities, and histopathological results were utilized in the present study. Multivariate logistic regression analysis demonstrated that mural nodules, spiculation, mural morphology, and the number of cystic cavities were independent factors for predicting benign and malignant cystic pulmonary nodules. The mural nodule showed increased solid components on the thin cystic wall. Current follow-up studies on subsolid pulmonary nodules have shown that the malignant degree and aggressiveness of such nodules with increasing solid component concentration [24–26]. In a previous follow-up study of 26 cystic lung cancer patients, all cystic lesions measuring 1 mm were found to be present after 12–118 months (median 35 months) from the initial CT scan [4]. Hence, mural nodules can become a key factor in determining the degree of tumor invasion [27]. Thus, cystic formation is a vital stage in the progression of solid lung cancer. Thus, mural nodule development can be considered a specific manifestation of cystic lung cancer and might be associated with the mechanism of cystic lung nodule formation. Among the many etiologies, the check valve mechanism has been widely recognized [20, 28]. In this theory, tumor cells invade the bronchus, cause local stenosis, and form a one-way living valve after originating from the alveolar and bronchial walls. Due to gas accumulation, the internal pressure increases, and the cystic airspace becomes larger. However, the growth of tumor tissue into the cystic cavity completely blocks the bronchus. Thus, the cavity’s solid component increases and the cystic airspace decreases. Furthermore, Shen et al. [14] discovered that cystic pulmonary cancer with mural nodules (type III) might be more invasive and have a worse prognosis. Type III tumors exhibit less differentiation than other morphologies (types I, II, and IV). However, when intermediate or low differentiation frequency reaches 85% [14], such patients exhibit low survival [29, 30]. Additionally, marginal features such as rough edges of malignant nodules, spiculation, and pleural tag signs are useful for diagnosing benign and malignant nodules. Speculation is a malignant feature characterized by distortion resulting from the infiltration of tumor cells into surrounding tissues. A higher degree of spiculation strongly indicates the possibility of malignant nodules; the incidence of spiculation in lung adenocarcinoma is 81–100% [31, 32]. Moreover, we reached the same conclusion that the spiculation sign was an independent risk factor for diagnosing cystic lung cancer. After evaluating the pathology of cystic pulmonary nodules, Tan et al.suggested that the irregular cystic margins observed on CT images were consistent with the fibrous tissue produced by tumor cells. However, benign lesions such as pulmonary bullae lack this feature, and their cystic walls are smooth [28]. Moreover, the multilocular septation observed on CT corresponds to fibrous tissues, airways, or vessels, indicating the possibility of malignancy. In malignant cystic pulmonary nodules, multilocular patterns with septation account for 58.4% of all cysts [28]. Thus, cystic pulmonary cancer might be correlated with a prior history of emphysema and bullae. Many studies have suggested that cystic pulmonary cancer might originate from cystic mural cells with preexisting pulmonary bullae; however, individuals with chronic obstructive pulmonary disease and emphysema might have a 4- to 5-fold increased risk of lung cancer [29, 33]. Moreover, in emphysema patients, cysts may interfere with ventilation and lung clearance, thereby leading to carcinogen deposition and resulting in malignancy [34]. Although our patients’ disease records included a history of bullae and emphysema, no significant difference was observed between the history of cystic lung cancer and our data analysis. This may be due to our exclusion criteria concerning those patients in whom the cystic cavities could not be distinguished from surrounding emphysema; this might have interfered with our results. Since the malignancy rate of pulmonary nodules is closely related to nodule diameter, the malignancy rate increases as nodule diameter increases [35]. However, whether the measurement of tumor diameter in cystic lung cancer patients should include cystic cavities is still uncertain [36–38]. The extent of cystic pulmonary nodule diameter on CT images is often greater than that determined by histopathology [14]. However, our study revealed no associations between the diameter of cystic pulmonary nodules or between benign and malignant nodules. This might be because our study’s diameter was the diameter of the cystic cavities rather than the diameter of the solid or ground glass components. Based on the use of low-dose thin-layer CT as an imaging method, our model displayed several advantages over traditional models, such as Mayo, VA, Brock, and PKUPH: (1) specific imaging features of cystic pulmonary nodules and the tumor marker CEA were added to create a comprehensive diagnostic model that combined patient history, tumor markers, and imaging data; (2) all vital risk factors in the nomogram were commonly available in clinical practice; and (3) our model’s ROC, calibration, and DCA curves were good, and its accuracy and reliability were satisfactory. Therefore, our proposed model can help clinicians foster more personalized risk predictions for each patient. This study has several limitations. First, cystic pulmonary nodule formation may be a crucial stage in the development of lung cancer. According to the literature, the median time between cystic cavity and lung cancer diagnosis is 25.5 months [39]. Therefore, the dynamic observation index of imaging is particularly important for diagnosing cystic cavity pulmonary nodules. Thus, the possibility of lung cancer should be considered when new mural nodules, solid components, or the density of mural nodules increases during follow-up. Surgical resection can be performed to confirm the diagnosis [28, 40]. Moreover, when the wall began to thicken irregularly or when wall nodules appeared, the proportion of wall attachment and acinous subtypes decreased according to histopathological findings. In contrast, the magnitude of the solid and micropapillary subtypes increased [20]. Hence, dynamic observation indicators such as the increase in solid components of the cystic wall and nodule density should be included in the later stages. Second, due to the small incidence of cystic cavity pulmonary nodules, our study data were procured from a single-center database. Therefore, our sample size was small, and there was a lack of data from other hospitals for model external verification. Thus, our conclusions may be biased and limit our predictive nomogram's generalizability. Therefore, future studies should increase the sample size by involving multiple centers and including sufficient samples to confirm our findings.

## Conclusion

To conclude, our model showed good predictive performance in evaluating probability of malignancy in patients with preoperative cystic pulmonary nodules. Moreover, this approach may provide surgeons with additional clinical reference information and diagnostic evidence for early intervention and timely treatment of cystic pulmonary nodules.

## Data Availability

No datasets were generated or analysed during the current study.

## Abbreviations

AUC: Area under the ROC curve
CEA: Carcinoembryonic antigen
CT: Computed tomography
DCA: Decision curve analysis
ROC: Receiver operating characteristic
SD: Standard Deviation
